# Proteomic insights into mycobacterial responses to elevated riboflavin reveal a role for flavin-sequestering proteins in flavin homeostasis

**DOI:** 10.3389/ftubr.2025.1719707

**Published:** 2025-12-17

**Authors:** Nurudeen Oketade, Karen M. Dobos

**Affiliations:** Department of Microbiology, Immunology and Pathology, Colorado State University, Fort Collins, CO, United States

**Keywords:** mycobacteria, riboflavin, flavin sequesters, proteomics, tuberculosis, isoniazid, homeostasis, stress response

## Abstract

Riboflavin biosynthesis is essential for most microorganisms, yet its production is tightly regulated due to the potential toxic effect of riboflavin and its derivatives. While regulatory mechanisms for this process have been described in other organisms, none has been identified in mycobacteria. Uncovering such a mechanism will be critical to fully exploit the riboflavin biosynthetic pathway as a pharmacological target. We previously observed that *Mycobacterium tuberculosis* (Mtb) and *Mycolicibacterium smegmatis* (Msm) can grow successfully under elevated riboflavin levels, prompting an investigation into how mycobacteria tolerates high intracellular riboflavin. Using a bioinformatic approach of proteomic data, we explored the global proteomic response of Mtb and Msm to elevated riboflavin. Our results revealed increased abundance of know flavin sequesters in response to high riboflavin levels, suggesting a role in flavin homeostasis. Using a transcription regulatory network analysis, we observed the employment of similar regulatory networks, most notably DosR, in both Mtb and Msm in response to elevated riboflavin. We also identified a potential link between the riboflavin induced response and isoniazid resistance mechanisms, warranting further investigation. Overall, this study provides evidence for the involvement of flavin sequesters in maintaining flavin homeostasis and highlights the potential regulatory role of DosR in this process.

## Introduction

Mtb, the causative organism of tuberculosis (TB) has established itself as an apex pathogen due to its remarkable ability to evade the defense mechanism of host and resist the bactericidal action of antimicrobials (1, 2). The dual capacity of immune evasion and antimicrobial resistance contributes to Mtb's aggressiveness during active infection and underlies the current complexity of TB treatment regimen, which requires prolonged combination therapy targeting distinct aspects of Mtb physiology (3). This has resulted in Mtb persisting as a global health burden. To effectively overcome the challenge posed by Mtb, a comprehensive understanding of Mtb's basic physiology is essential.

The riboflavin biosynthesis pathway is a highly conserved and an essential component of mycobacterial physiology (4, 5). Flavin mononucleotide (FMN) and flavin adenine dinucleotide (FAD) and their respective redox couples FMNH_2_ and FADH_2_ serve a multitude of functions which includes respiration, redox homeostasis, lipid metabolism, and cellular signaling in mycobacteria (6). In addition to producing FMN and FAD, this pathway is also involved in the production of metabolic antigens recognized by MR1T cells (5, 7). In other bacteria, the synthesis and abundance of these cofactors has been shown to be tightly regulated. In several bacterial species including *B. subtilis* and *E.coli*, the synthesis of riboflavin is controlled by an FMN dependent riboswitch through a negative feedback mechanism (8). In other microorganisms, the level of intracellular riboflavin can be controlled through balance in an active efflux and influx (8, 9). In yeast, RibA2, the enzyme that catalyzes the hydrolyzation of GTP, the rate limiting step of the pathway, is allosterically inhibited by FAD (8). Allosteric inhibition of RibF, a flavin kinase, by FMN and FAD, has also been reported in other bacteria (8). The need for this tight regulation of riboflavin biosynthesis has been attributed to the potential toxic effect of riboflavin, FMN and FAD to cells via autooxidation (10). Although, the toxic effect of riboflavin has mostly been attributed to the generation of reactive oxygen species (ROS) during photoactivation (10), there have also been reports of direct riboflavin toxicity to microorganisms. High levels of riboflavin have been reported to be bactericidal in *Staphylococcus aureus, Enterococcus faecalis, Salmonella typhi*, and *Pseudomonas aeruginosa* (11–13). It is hypothesized that elevated riboflavin levels might exert these toxic effects by generating reactive oxygen species (ROS) via autooxidation and disrupting the activity of flavin-dependent enzymes, as demonstrated in a study on its antifungal effects in *Cryptococcus neoformans* (14).

Given the stringent regulation of riboflavin biosynthesis observed in other species, it has been hypothesized that similar regulatory mechanisms may operate in mycobacteria. To date, no riboswitch regulating the riboflavin biosynthetic pathway (RBP) has been identified in mycobacteria, with bioinformatic analyses confirming its absence (15). Additionally, no flavin efflux system has been detected in mycobacteria either experimentally or bioinformatically (16, 17). While allosteric inhibition of RibA2 and RibF has been described in yeast and other bacteria, respectively, such regulation has not been observed in mycobacteria. Moreover, allosteric inhibition of RibA2 in mycobacteria appears unlikely, as elevated riboflavin levels do not affect MR1 antigenicity, a process dependent on the production of 5-A-RU by RibA2 (5). Although the absence of known regulatory mechanisms for flavin biosynthesis suggests that mycobacteria may not rely on conventional feedback control, other mechanisms likely exist and remain poorly understood. Two potential mechanisms have been hypothesized to regulate intracellular flavin levels: the redox homeostatic system (RHOCS) (18) and flavin sequestering (19–21). RHOCS has been implicated in the degradation of FAD and NAD(P)H to maintain redox homeostasis; however, it is primarily activated in response to NAD(P)H, suggesting a broader role in redox homeostasis rather than direct control of flavin availability. Similarly, while flavin sequestering has been associated with redox homeostasis, a direct link to the regulation of intracellular flavin concentrations has yet to be established.

In this study, we use a bioinformatic approach to investigate the proteomic alterations in mycobacteria following exposure to elevated levels of exogenous riboflavin. Using a data-independent acquisition (DIA)-Parallel Accumulation Serial Fragmentation (PASEF) based untargeted proteomics approach, we analyzed the Mtb and Msm cultured with or without riboflavin supplementation during early logarithmic growth. The objective was to gain insight into how mycobacteria respond to elevated intracellular riboflavin levels and to compare the responses between Mtb, a pathogenic species, and Msm, a non-pathogenic environmental mycobacterium. Both univariate and multivariate analyses revealed coordinated proteomic changes in response to high riboflavin levels in both species, although the response was less pronounced in Mtb. Mainly, we observed an increased abundance of known flavin sequesters including dodecin, Fsq, and Acg in both Mtb and Msm following riboflavin supplementation. We also saw a general upregulation of DosR regulatory targets in response to elevated riboflavin indicating a regulatory network response for flavin homeostasis. Additionally, we observed a consistent increase in OxiR, a TetR/AcrR family transcription factor implicated in isoniazid (INH) resistance, in both species. In contrast, other INH resistance associated proteins: IniA, IniB, and IniC showed reduced abundance only in Mtb. Opposite to the trend seen with OxiR, levels of PpiA, a secreted protein linked to stress adaptation, were decreased in both Mtb and Msm. Together, these findings suggest that flavin sequestration and stress adaptation may play key roles in maintaining flavin homeostasis in mycobacteria. Also, our findings point toward a link between flavin homeostasis and susceptibility to INH underscoring the need for further analysis.

## Materials and methods

### Growth and preparation of Msm and Mtb

Glycerol stocks of Mtb and Msm strains were used to start cultures on 7H11 agar plates supplemented with or without riboflavin (22 μM for Mtb and 85 μM for Msm). Plates were incubated for 2 weeks for Mtb and 4–5 days Msm at 37 °C. Cells were scraped from plates into 7H9 media supplemented with OADC with or without riboflavin and grown at 37 °C until mid-log phase (22 μM for Mtb and 85 μM for Msm). Cells were collected via centrifugation, washed with mass spectrometry grade water and resuspended in Acetonitrile/Methanol/Water (2:2:1) and then transferred to homogenization tubes prefilled with 0.1 mm zircon beads. The cells were homogenized at 6 m/s for 30 s (10 rounds) with intermittent cooling on ice for 2 min. Homogenized cells were spun at 16,000 g for 20 min and supernatant collected for metabolomics and pelleted cell lysate collected for proteomics. Proteins were quantified using Bicinchoninic acid assay (Thermo Scientific, Cat No.: 23225). Supernatant and pellet were then stored at −20°C until further processed (5).

### LC-MS/MS analysis (proteomics)

Thirty μg of pelleted protein from Msm and Mtb was subjected to in-gel trypsin digestion as described previously (23). Digested proteins were resuspended in loading buffer (3% acetonitrile, 0.1% formic acid in water) to achieve a final concentration of 250 ng/μl. All samples were analyzed using nano-UHPLC (ultrahigh-pressure liquid chromatography) nanoElute (Bruker Daltonics, USA) coupled to a timsTOF Pro (Bruker Daltonics, USA) mass spectrometer. One micro liter of total peptides were loaded onto a trap cartridge using PepMap Neo Trap Cartridge (Thermo Scientific, Cat: 174500) (5 μm C18 300 μm × 5 mm) packed with spherical, fully porous, ultrapure silica and reverse phase UHPLC was performed in a PepSep Column (Bruker Daltonics, Part No:1895802) (1.5 μm C18 75 μm × 25 cm). Mobile phase A was 0.1% formic acid in water; mobile phase B was 0.1% formic acid in acetonitrile. Liquid chromatography (LC) separation was carried out at a flow rate of 0.5 μL/min using a linear gradient of 5% solvent B to 30% solvent B over a duration 17.8 min, then 30% solvent B to 95% solvent B for 2.5 min. Thereafter, 95% solvent B was held for 2.4 min for equilibration.

Mass spectra and tandem mass spectra were obtained using the parallel accumulation serial fragmentation (PASEF) acquisition method in positive ion mode. Captive spray source was operated with the following settings: 1,600 V capillary voltage, 3.0 l/min of dry gas at 200 °C with the NanoBooster switched off.

For DIA-PASEF analysis, data was acquired with a method consisting of 24 mass steps per cycle (Supplementary Table S1). Mass width was set at 50 Da with 1 Da overlap between windows covering a mass range between 307.8 to 1484.8 Da. The ion mobility range (1/K0) covered was between 0.71 and 1.44. The collision energy was set to follow a linear function starting from 20 eV for 0.6 V. s/cm^2^ to 59 eV for 1.6 V. s/cm^2^.

### Data analysis of DIA-PASEF

DIA-PASEF data was processed on FragPipe software v. 19.1 (MSFragger version 3.7, IonQuant version 1.8.10, Philosopher version 4.8.1) using the DIA_SpecLib-Quant workflow as described (24). DIA raw files were loaded with DIA Quant selected as data type. A search was conducted using default settings with the FASTA sequence obtained from the Mycobrowser database (Release 4, 2021-03-23). Decoy and contaminant sequences were generated by FragPipe. Default settings of MSFragger was used, including oxidation of M (+ 15.9949 Da mass shift) acetylation at N-terminal (+ 42.0106) as variable modifications. Advanced output options were set to write calibrated mzML and MGF files. Tryptic searches were performed with 20 ppm precursor and fragment tolerance permitting up to 2 missed cleavages. Search results were filtered to 1% FDR at both protein and peptide levels using peptide prophet. Output files with quantification based on summed intensity of all ions for a single peptide and summed intensity of peptides for a single protein were generated. The output file containing summed intensity of peptides for a single protein was used for quantification of proteins and is provided in Supplementary material. The raw mass spectrometry proteomics data have been deposited to the ProteomeXchange Consortium via the PRIDE (22) partner repository with the dataset identifiers PXD06833 (Mtb) and PXD068360 (Msm).

### LC-MS/MS analysis (metabolomics)

Samples collected for metabolomics was dried in savant and their weight determined. Dried samples were then resuspended in 3% Acetonitrile in Water (0.1% Formic Acid) to make a final concentration of 2 mg/ml for Mtb and 10 mg/ml for Msm. 2 μl of resuspended samples were loaded per injection. Reverse phase chromatography was performed using the InfinityLab Poroshell 120 EC-C8 Column (Agilent, Part Number:) (2.1 × 100 mm, 2.7 μm) on the using the Agilent 1290 Infinity III Liquid Chromatography System. Mobile phase A was 0.1% formic acid in water; mobile phase B was 0.1% formic acid in acetonitrile. LC separation was carried out at a flow rate of 0.32 μl/min using a linear gradient of 2% solvent B held for 3 min and then to 50% solvent B over a duration of 15 min, then 50% solvent B to 99% solvent B over 7 min. 99% solvent B was held for 6.5 min and then returned to 2% solvent B for 1.5 min and held for 5 min for equilibration. Sample eluted within the first minute was diverted to waste to prevent clogging.

MS^1^ and MS^2^ scans were obtained in positive ion mode. Electrospray ionization source (ESI) of the Agilent 6546 quadrupole time-of-flight (QTOF) was operated with the following settings: 3,500 capillary voltage, 10.0 l/min of dry gas at 40 psi and 320 °C. Post MS-inlet capillary, fragmentor was set to 120 V, nozzle voltage was set to 1,000 V, and skimmer voltage was set to 40 V. The peak-to-peak voltage of the ion transfer octupole was set at 750 V. The mass analyzers for MS and MS/MS were set to scan between *m/z* 100 and 1,700 at a scan rate of 4 spectra/s and an isolation width of ≈1.3 amu. 3 precursors were selected per cycle with an absolute threshold of 200 and relative threshold of 0.01%. Mass error tolerance was set to ± 20 ppm and retention time exclusion tolerance set to ± 0.2 min. Collision energy was alternated between 15- and 30-volts during cycle.

For riboflavin, FMN and FAD, standards were purchased from Sigma-Alrich (Riboflavin, Cat No.: R4500) and Cayman Chemical Company (FMN, Cat No.: 18167; FAD, Cat No.: 23386)0.1 μg/ml of standards were analyzed by LC-MS/MS as described to determine the determine their retention time (RT), *m/z* and collision induced dissociation (CID) product ion spectrum. For samples from Mtb and Msm, Q-TOF was set to targeted MS/MS with precursor *m/z*, RT, RT window (1.5 min), isolation width (~1.3 m/z), charged state (+1) set for riboflavin, FMN and FAD provided as a targeted list for analysis. MS^1^ extracted ion chromatogram (EIC) obtained through Agilent MassHunter Qualitative Analysis version 10.0 was used for each the quantification of each metabolite. MS^2^ spectra for compounds were inspected and compared to synthetic standard to confirm identification. Details of precursor *m/z*, transitions *m/*z, RT for riboflavin, FMN and FAD are provided in Supplementary Table S2. Raw data from targeted metabolomics have been deposited at the Dryad Digital Repository and are available at: doi: 10.5061/dryad.pg4f4qs39

### Bioinformatic and statistical analyses

Univariate analysis was performed using GraphPad prism 10 statistical software (GraphPad Software, Inc., San Diego, CA) for metabolomics data. Statistical comparisons were performed using an unpaired *t*-test. Univariate and multivariate analyses were performed using R Studio for proteomics data. Prior to analyzing the proteomic data, single protein intensity for proteins obtained from FragPipe was checked for missingness for Mtb (Supplementary Figure S1A) and Msm (Supplementary Figure S2A). For proteins with less than 50% missing abundance values per sample, a sample-specific imputation strategy was employed, whereby missing values were estimated using data from the corresponding biological and technical replicates (Supplementary Figures S1B, S2B). Proteins with more that 50% missingness, missing values were replaced with one-fifth of observed minimum intensity. Following this process, 2,725 proteins were confidently detected in Mtb (Supplementary Figure S1B) and 3,917 in Msm (Supplementary Figure S2B). Biological and technical coefficient of variation was also evaluated to ensure precision and data quality (Supplementary Figures S1D, E, S2D, E). Intensities of retained proteins were then log2 transformed prior to analyses. For univariate analysis, the *limma* software package (25) in R studio was used to compare the mean of proteins between the two samples using an unpaired *t*-test with *p*-values adjusted for multiple comparison test using the Benjamini–Hochberg method. Comparisons were considered differentially abundant if *p* < 0.05 and log2 fold change > 1. For multivariate analysis, the *mixomics* package (26) in R studio was used to carry out sparse Partial Least-Squares Discriminant Analysis (sPLS-DA). Log2 transformed intensities were centered and scaled prior to sPLS-DA analysis. For feature selection, the sPLS-DA model was tuned as described in the tutorial provided and eventually set at 3 components with 100 features per component. The 100 most discriminant proteins between groups from component 1 were selected for further analysis. Functional protein association network analysis was carried out for limma and sPLS-DA selected features in STRING (version 12.0) (27) to analyze protein-protein-interaction (PPI) for features selected by both univariate and multivariate analysis. Transcription Factor Over Expression (TFOE) Dataset from the Environment-Specific Gene Regulatory Network for Mtb (ERGIN) was used to identify TFs that may be involved in the response to elevated intracellular riboflavin (28). Flavin dependent proteins from Mtb and Msm were identified from the UniProtKB database based on their annotation as an FMN or FAD dependent/binding protein. Data used for analyses are provided as Supplementary Table S3.

## Results

### Elevated intracellular riboflavin does not increase downstream flavin pools in mycobacteria

**Figure 1:**
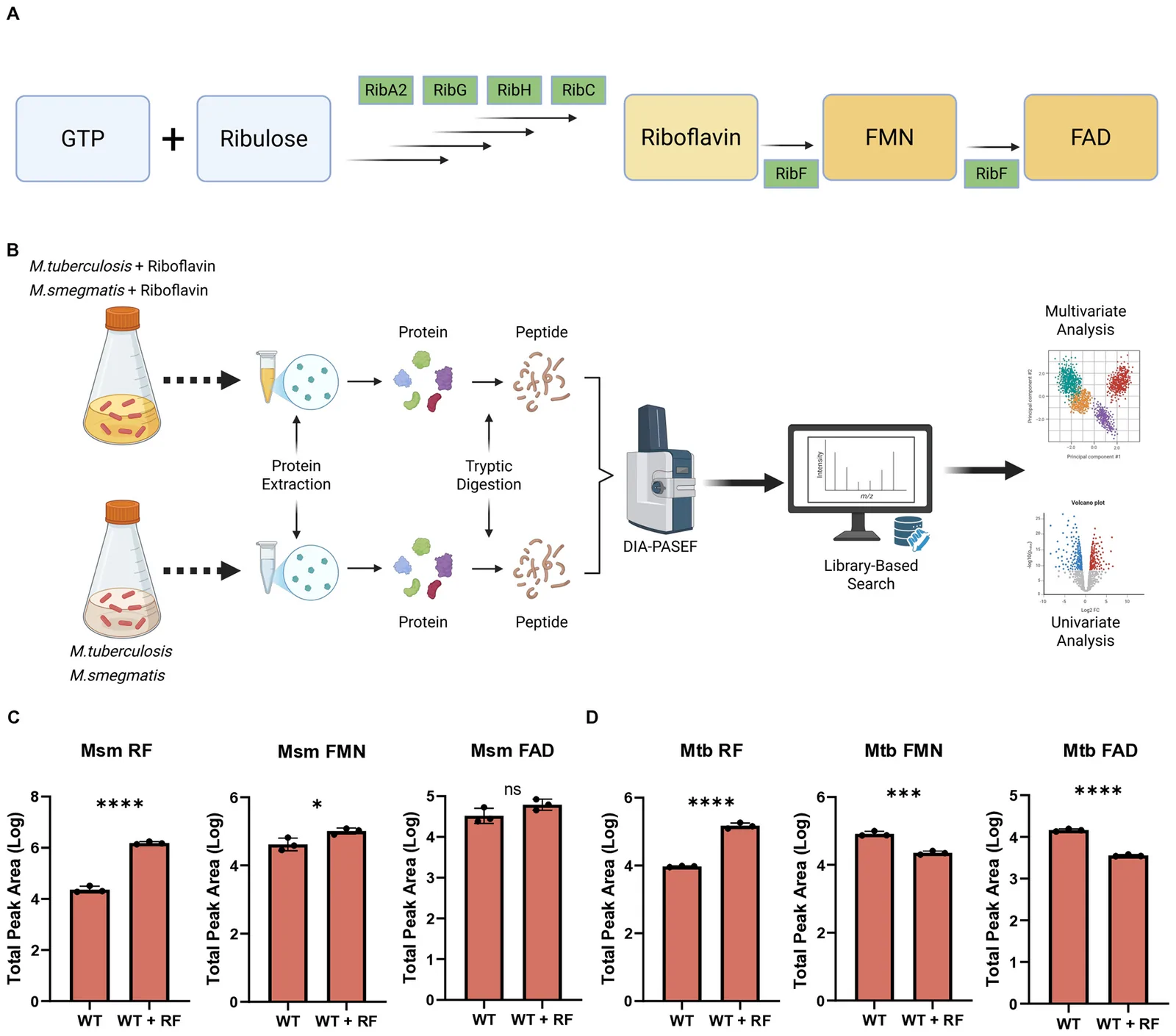
**(A)** Overview of riboflavin biosynthetic pathway. Large boxes indicate intermediates or final product of pathway while small boxes indicate enzyme. **(B)** Schematic representation of experimental workflow used to profile proteome in response to elevated riboflavin. **(C, D)** Levels of intracellular riboflavin, FMN and FAD in Msm **(C)** and Mtb **(D)** measured using HRMS. Data are shown as mean quantification ± SEM for three biological replicates. Quantification was carried out using extracted ion chromatogram (EIC) peak area. Statistical comparisons were performed using an unpaired *t*-test whereby statistical significance is represented by *p* < 0.05, *p* < 0.0005, *p* < 0.0001, shown by *, ***, ****, respectively. GTP, guanosine triphosphate; FMN, flavin mononucleotide; FAD, flavin adenine dinucleotide; WT, Wild type; RF, riboflavin; ns, not significant. Created with BioRender.com.

Previous work has shown that elevated intracellular levels of riboflavin did not affect the growth rate of mycobacteria (4, 5). Additionally, we reported that riboflavin abundance had no significant impact on the expression levels of RBP proteins, with the exception of RibF, which was not included in the prior dataset (5). In our previous study, we observed markedly elevated intracellular riboflavin levels in both Mtb and Msm following supplementation, with concentrations reaching up to 100-fold higher than those produced endogenously. Riboflavin is phosphorylated to FMN and further adenylated to FAD by RibF, with both cofactors supporting a range of flavin-dependent enzymatic processes (Figure 1A). To assess whether elevated riboflavin levels affected RibF abundance, we compared its levels in riboflavin-treated and untreated samples. We observed an increase in RibF abundance under elevated riboflavin conditions in both Msm and Mtb (Supplementary Figures S3A, B). Next, we assessed if elevated riboflavin led to increased synthesis of its downstream products, FMN and FAD. To achieve this, we utilized LC-MS/MS based metabolomics to assess the levels of downstream flavin metabolites. Using High Resolution Mass Spectrometry (HRMS), we quantified riboflavin, FMN and FAD in Mtb and Msm cultured with or without exogenous riboflavin (Figures 1C, D, Supplementary Figures S3–S5). Same as our previous result, we observed a 2-log increase in intracellular riboflavin between the samples for both Msm and Mtb. Interestingly, the intracellular levels of both FMN and FAD did not recapitulate what was observed for riboflavin. In Msm, the levels of FAD and FMN were similar in both riboflavin treated and untreated samples with a slight increase in FMN. In Mtb, the levels of FMN and FAD were lower upon treatment with riboflavin.

### Elevated intracellular riboflavin increases the abundance of flavin-sequestering proteins

**Figure 2:**
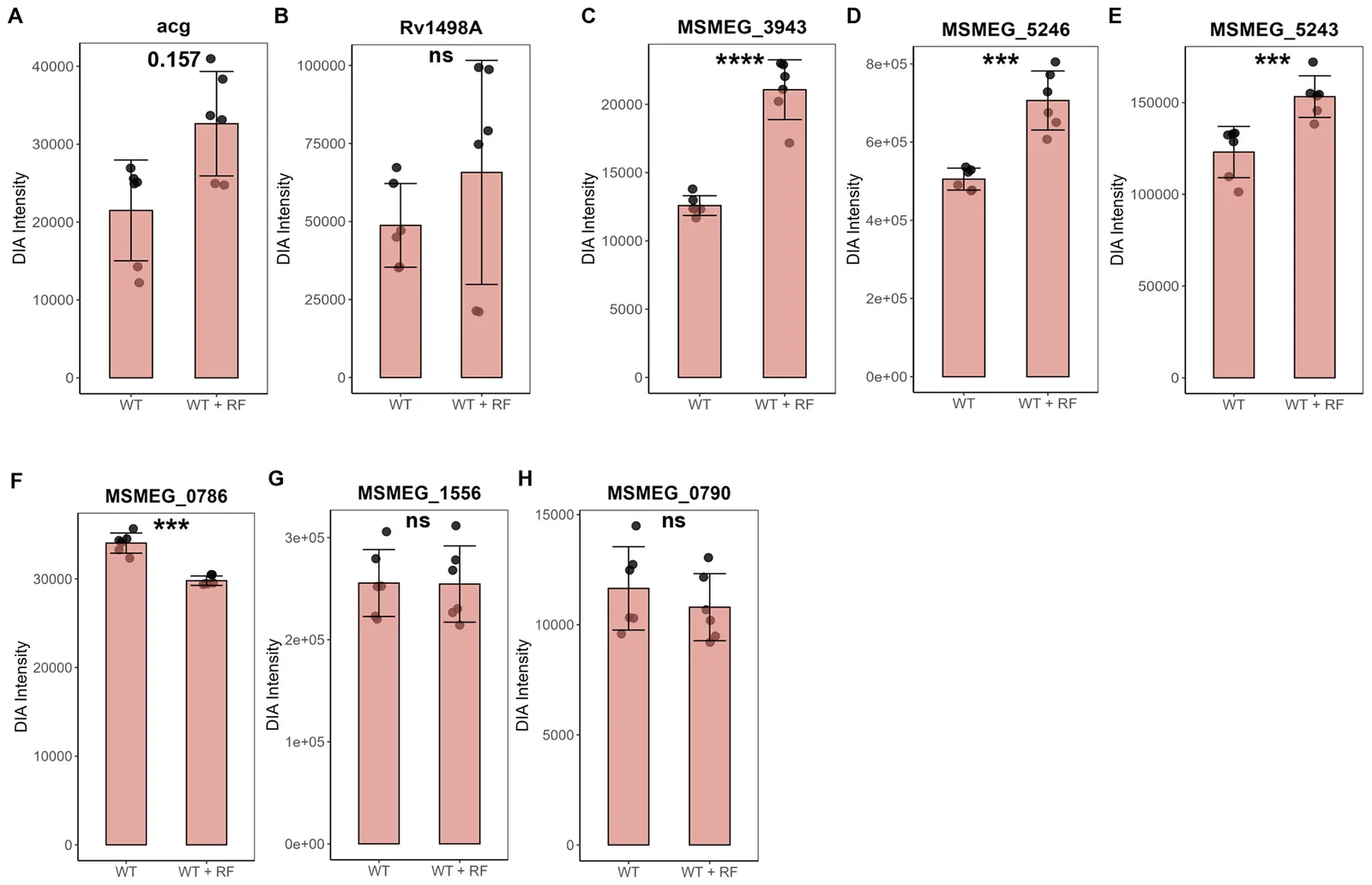
Impact of elevated intracellular riboflavin on abundance of flavin sequesters in Msm and Mtb. **(A–H)** Protein abundance was measured using DIA-PASEF proteomics (*n* = 3 biological replicates, 2 technical replicates). Elevated intracellular riboflavin increases abundance of flavin sequesters in Mtb and Msm. Mtb Acg **(A)**, Mtb Dodecin **(B)**, Msm Acg **(C, D)**, and Msm Fsq **(E)**. Elevated Intracellular riboflavin levels did not increase abundance of RHOCS protein members in Msm. PknG **(F)**, RenU **(G)** and L13 **(H)**. Data are shown as mean quantification ± SD. Statistical comparison was performed using log transformed values followed by unpaired *t*-test and multiple test correction using Benjamini…Hochberg adjusted *p*-value. Statistical significance is represented by *p* < 0.0005, *p* < 0.0001, shown by ***, ****, respectively. WT, Wild type; RF, riboflavin; ns, not significant.

RHOCs and flavin sequesters have been proposed to play a role in contributing to flavin homeostasis in Mtb and Msm (18, 19, 21). To investigate their involvement, we examined whether their abundance is altered in response to elevated intracellular riboflavin levels (Figure 1B). For this analysis, we leveraged previously acquired data from LC-MS/MS based Data Independent Acquisition Parallel Acquisition and Serial Fragmentation (DIA-PASEF) proteomic profiling on Msm and Mtb cultured with or without exogenous riboflavin. Using this dataset, we assessed the impact of elevated intracellular riboflavin on the abundance of known flavin sequesters (Figures 2A–E) and RHOCS protein members (Figures 2F–H). Acg, Acr co-regulated gene (Rv2032), and its homologs in Msm (MSMEG_3943, MSMEG_5246), have been described as flavin sequesters in both Msm and Mtb (29), whereas dodecin (Rv1498A) and fsq (MSMEG_5243) serve similar functions in Mtb and Msm. respectively (19, 21). For Acg, we observed an increase in its abundance in both Mtb (Figure 2A) and Msm (Figures 2C, D) in response to elevated intracellular riboflavin, with a more pronounced response in Msm. Dodecin and Fsq showed similar abundance patterns to Acg in Mtb (Figure 2B) and Msm (Figure 2E), respectively. Among RHOCS protein members which include RenU (MSMEG_0790) and L13 (MSMEG_1556), no change in abundance was observed in Msm upon exposure to exogenous riboflavin (Figures 2F, G), except for PknG (MSMEG_0786) which showed a decrease (Figure 2H). In contrast to Msm, none of the RHOCS member proteins (RenU: Rv0413, L13: Rv3443c, and PknG:Rv0410c) were detected in our dataset for Mtb.

### Mtb and Msm reprograms proteome in response to elevated intracellular riboflavin

**Figure 3:**
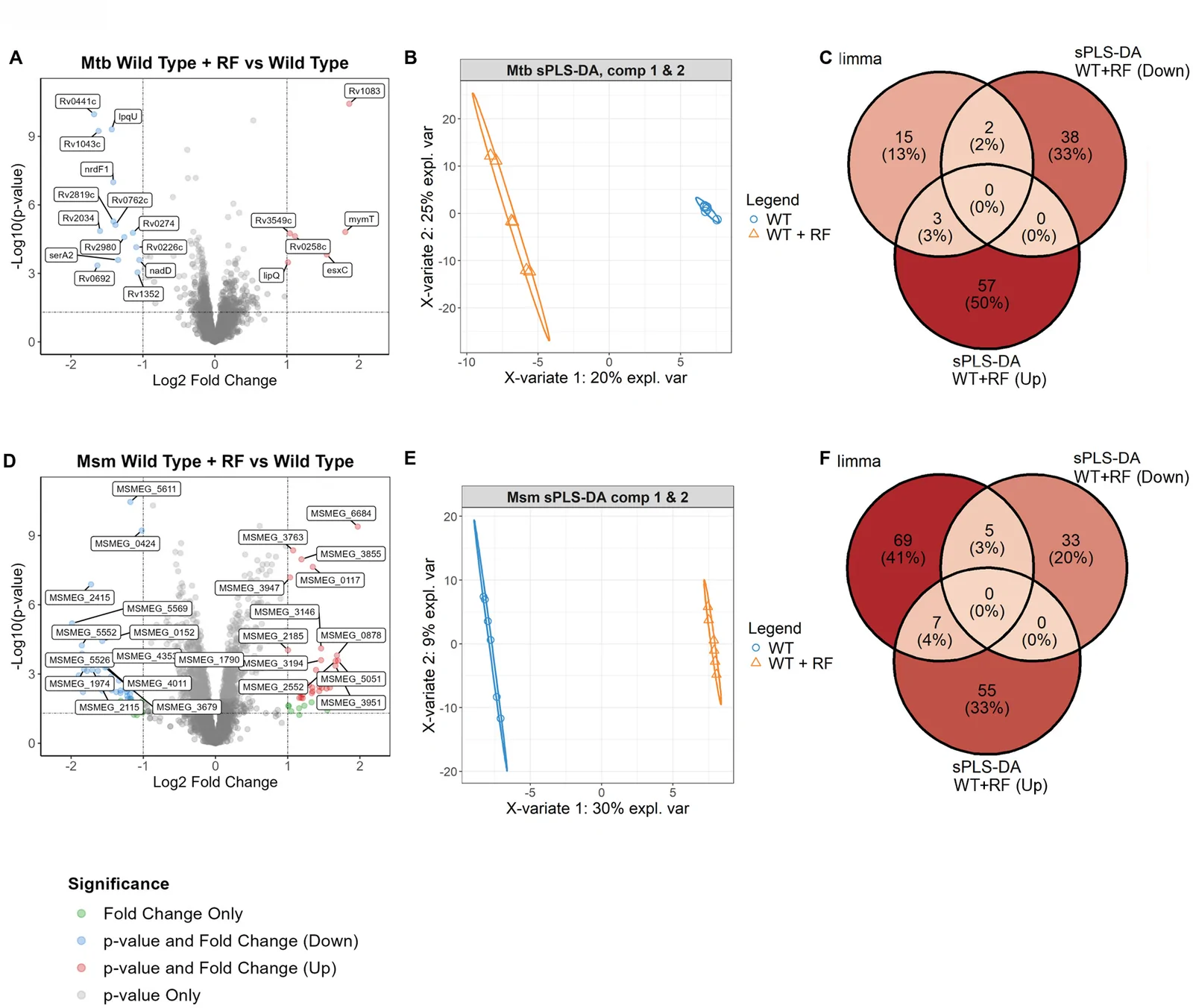
Elevated intracellular riboflavin induces proteome-wide change in Mtb and Msm. Proteome-wide response to elevated intracellular riboflavin in Mtb **(A, B)** and Msm **(D, E)**. Volcano plots **(A, D)** showing –log10 *p*-value vs. log2 fold change of riboflavin treated cells to untreated cells. Statistical comparison was performed using log transformed values followed by unpaired *t*-test and multiple test correction using Benjamini–Hochberg adjusted *p*-value. Mean fold-change across 3 biological replicates and two technical replicates are shown. Horizontal and vertical lines showing corresponding *p*-value at 0.05 and log2 fold change at 1. Positive log2 fold-change values can be interpreted as having a greater abundance in Wild Type + Riboflavin. Sparse Partial Least Square Analysis (sPLS-DA) plot **(D, E)** showing separation of riboflavin treated and untreated samples (*n* = 3 biological replicates, 2 technical replicates) based on 100 features per component. Overlap between features selected using univariate analysis cutoff (*p*-value <0.05 and log2 Fold change >1) and first principal component of sPLS-DA for Mtb **(C)** and Msm **(F)**. WT, Wild type; RF, riboflavin.

Since some of the flavin sequesters were shown to be part of the DosR stress response regulon (21), we hypothesized that Mtb and Msm may respond to elevated intracellular riboflavin through other proteins beyond the flavin sequesters. To identify other proteins associated with response to high intracellular riboflavin, we further analyzed the whole-proteome dataset generated by DIA-PASEF proteomics. Our goal was to detect differentially abundant proteins and identify those proteins capable of discriminating between treated and untreated samples. Statistical analysis was performed using the *limma* package in R to determine differentially abundant proteins between riboflavin-treated and untreated samples (Figure 3). In parallel, the *mixOmics* package in R was used to perform Sparse Partial Least Squares Discriminant Analysis (sPLS-DA) allowing us to classify samples and select proteins with a similar abundance pattern that could distinguish the two treatment groups (Figure 3). Using *limma*, we identified 20 and 81 proteins as differentially abundant in Mtb (Figure 3A) and Msm (Figure 3D), respectively, based on an adjusted *p*-value <0.05 and an absolute log2 fold change >1. We then carried out a STRING analysis to understand the physiological relevance of differentially abundant proteins (Supplementary Figures S7A, B). For Mtb (Supplementary Figure S7A), there was no enrichment likely due to the low number of proteins. However, we observed a distinct network formed by secretory proteins, EsxC, EsxD and EspG2. The rest of the proteins were of putative or unknown function. For Msm (Supplementary Figure S7B), similar to Mtb, majority of the proteins were also of unknown function. However, 5 of the proteins were annotated as bacterial regulatory proteins of the tetR family. We also observed a distinct network around eccA3 (MSMEG_0615), the cytosolic component of the esx-3 secretion system involved in siderophore-mediated iron acquisition.

The sPLS-DA model revealed clear separation between treatment groups in both Mtb and Msm based on first principal component (Figures 3B, E). For Mtb, the first component accounted for 20% of the explained variance (Figure 3B). In Msm, the first component explained 30% of the variance (Figure 3E). We then used the top 100 features based on their loading score on the first component from the sPLS-DA model to generate heatmaps for Mtb (Supplementary Figures S8A, B) and Msm (Supplementary Figures S8D, E), highlighting distinct proteins' abundance profiles in response to riboflavin in both organisms. In both Mtb and Msm, most of the selected proteins were involved in intermediate metabolism and respiration, followed by cell wall and cell processes (Supplementary Figures S8G, H). We also queried the proteins selected by sPLS-DA via STRING analysis to understand their biological relevance (Supplementary Figures S7C–H). For Mtb, most of the identified proteins were associated with the membrane. A distinct subnetwork was formed by components of the cytochrome complex, while another cluster comprised dehydrogenases, reductases, and fatty acid synthases positioned at the core of the network. Several proteins were not connected to any major cluster and appeared as isolated nodes. In addition, no distinct functional category was enriched indicating that the proteins selected by sPLS-DA for Mtb were functionally diverse. For Msm, the proteins selected by sPLS-DA were categorized to be involved in response to stress, hypoxia and chemicals for biological processes while 10 different molecular functions were enriched for including small molecule binding among others.

To assess concordance between the univariate and multivariate approaches, we compared the proteins identified as differentially abundant by *limma* with those selected by the sPLS-DA model. In total, 5 proteins in Mtb (Figure 3C) and 12 proteins in Msm (Figure 3F) were identified by both *limma* and sPLS-DA. The limited overlap suggests that proteins contributing to sample discrimination in the multivariate model may exhibit more subtle abundance changes that fall below the thresholds applied in our univariate analysis. We also considered if the proteomic changes were majorly seen in flavin dependent enzymes. Only 5 and 7 proteins that were either differentially abundant or selected by sPLS-DA were flavin dependent in Mtb and Msm, respectively, according to the UniprotKB database (Supplementary Figures S8C, F).

### Stress response regulatory networks drive the response to elevated riboflavin in mycobacteria

**Figure 4:**
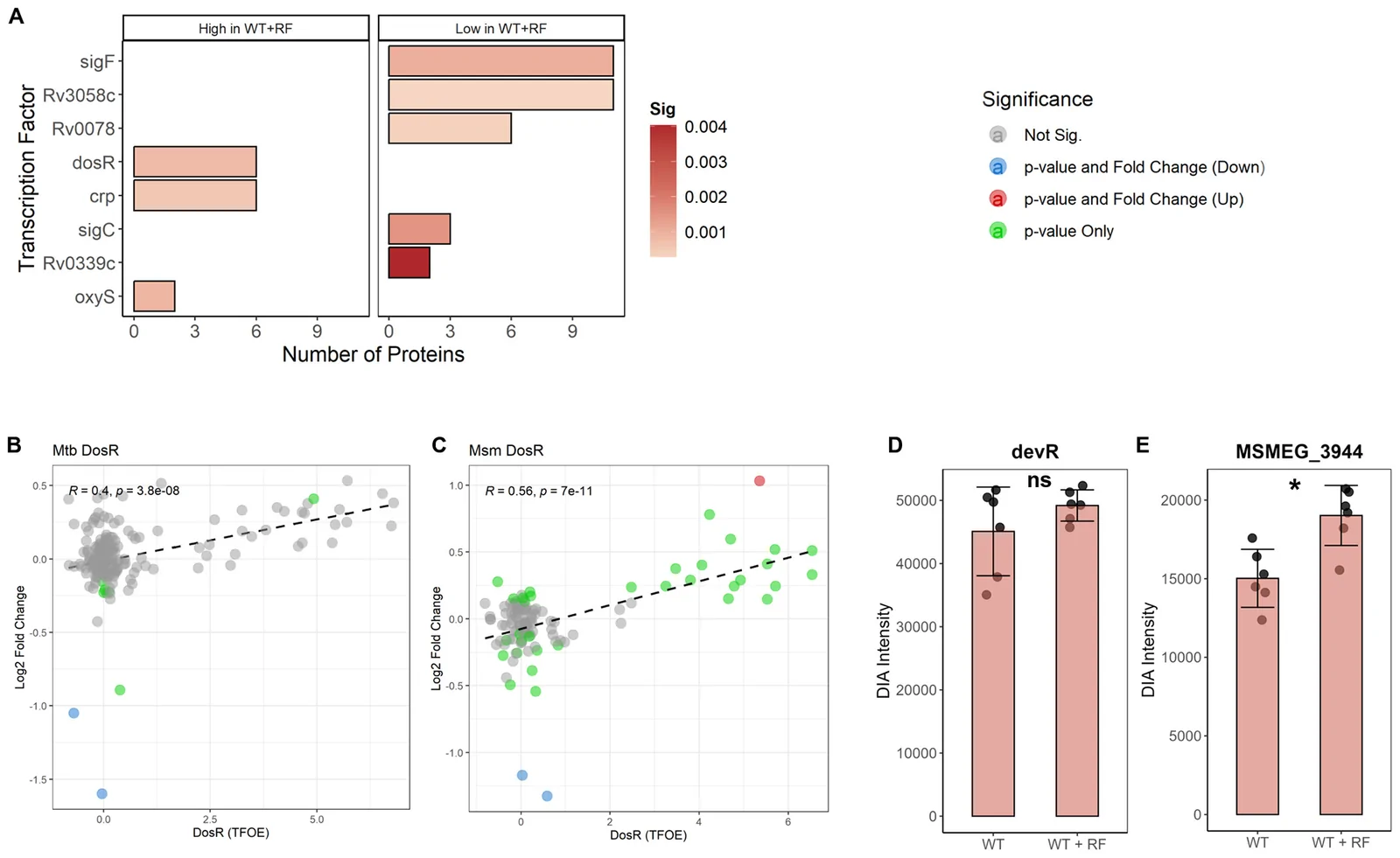
Transcription Factor Profiling Reveals DosR-Associated Proteome-Wide Changes in Response to Elevated Intracellular Riboflavin in Msm. **(A)** Features selected by sPLS-DA were assessed using ERGIN Transcription factor overexpression (TFOE) dataset to determine major transcription factors involved in response in Mtb. Left and right boxes shows number of features regulated by transcription factor downregulated and upregulated in riboflavin treated samples, respectively. The *p*-value indicates significance of overlap between sPLS-DA selected features and proteins regulated by transcription factor. **(B, C)** Pearson correlation between log2 fold change and TFOE expression change for regulatory targets (direct and operonic) of transcription factors based on ERGIN ChIP-seq data. Correlation coefficient R and corresponding *p*-value shown. A significant correlation is observed for DosR (positive) in Mtb **(B)** and Msm **(C)**. Legend showing statistical significance of fold change based on cut-off for univariate analysis (*p*-value <0.05 and log2 Fold change >1). **(D, E)** Protein abundance was measured using DIA-PASEF proteomics (*n* = 3 biological replicates, 2 technical replicates). Elevated intracellular riboflavin increases abundance of DosR in Msm but not Mtb. Msm DosR **(D)**, Mtb DosR **(E)**. Data are shown as mean quantification ± SD. Statistical comparison was performed using log transformed values followed by unpaired *t*-test and multiple test correction using Benjamini–Hochberg adjusted *p*-value. Statistical significance is represented by *p* < 0.05, shown by *, respectively. WT, Wild type; RF, riboflavin; ns/Not Sig., not significant.

Using the Transcription Factor Overexpression (TFOE) network provided by the Environmental Gene Regulatory Influence Network (EGRIN) for Mtb (28), we sought to identify transcriptional regulators that may underlie the proteomic patterns revealed by our sPLS-DA model in response to riboflavin. Among the 40 proteins with increased abundance in riboflavin-treated cells compared to untreated controls, 6 were regulated by Crp, 6 by DosR, and 2 by OxyS (Figure 4A). On the other hand, among the 60 proteins with decreased abundance, 11 were regulated by Rv3058c, 6 by Rv0078, 11 by SigF, 2 by Rv0339c and 3 by SigC (Figure 4A). To further explore the influence of these transcription factors (TFs) on the response we observed, we leveraged the ChIP-seq data from ERGIN to identify proteins that are direct regulatory targets of the aforementioned TFs. We then compared the Log2 fold change from our proteomic dataset with their corresponding TFOE response to determine if these proteins followed the same regulatory pattern as predicted by ERGIN (Figures 4B, C and Supplementary Figure S9). Rv3058 and Rv0078 both had one and two direct regulatory targets, respectively, and were excluded from the analysis. For DosR (Figure 4B), SigC (Supplementary Figure S9B), and OxyS (Supplementary Figure S9E), we observed a significant correlation between the TFOE response and the fold change in riboflavin-treated samples, with DosR and OxyS showing positive correlations and SigC showing a negative correlation. Only a small subset of their regulatory targets exhibited statistically significant fold changes. Nevertheless, the strength of the correlations and the agreement in the direction of the trends with the observed fold changes suggested a potential role for these transcription factors in the response to elevated riboflavin. We hypothesized that the TFOE data for Mtb might reveal similar trends in responses for TFs that are conserved in Msm. To sanction this approach, we first checked for the presence of homologs for these TFs in Msm. Out of the six TFs that we assesses in Mtb, we identified 5 that had homologs in Msm (SigF: MSMEG_1804, Rv0339: MSMEG_0691, Crp: Rv3676, OxyS: MSMEG_0156, DosR: MSMEG_3944) with the exception being SigC (30). We then looked at the correlation between TFOE response and the fold change in Msm (Figure 4 and Supplementary Figure S9). Similar to Mtb, we observed no significant correlation for the other TFs (Supplementary Figures S9F–H). However, we observed a significant correlation for DosR (Figure 4C) and OxyS (Supplementary Figure S9I). Converse to what we observed in Mtb, there was a negative correlation between TFOE response and fold change for OxyS, although none of the regulatory targets had a statistically significant fold change. For the regulatory targets of DosR, similar to Mtb, there was a positive correlation between the fold change and TFOE response. However, for Msm most of the regulatory targets of DosR had a statistically significant fold change. Given the consistency in the regulatory pattern of the DosR regulatory targets in both organisms, albeit stronger in Msm, we examined whether DosR abundance itself was altered in response to riboflavin (Figures 4D, E). In Msm (MSMEG_3944), DosR levels increased significantly (Figure 4E), whereas in Mtb, there was no increase (Figure 4D). This lack of change in DosR abundance in Mtb aligns with the broader differences in the proteomic response to elevated riboflavin observed between the two species.

### Limited convergence in Mtb and Msm response to elevated intracellular riboflavin

**Figure 5:**
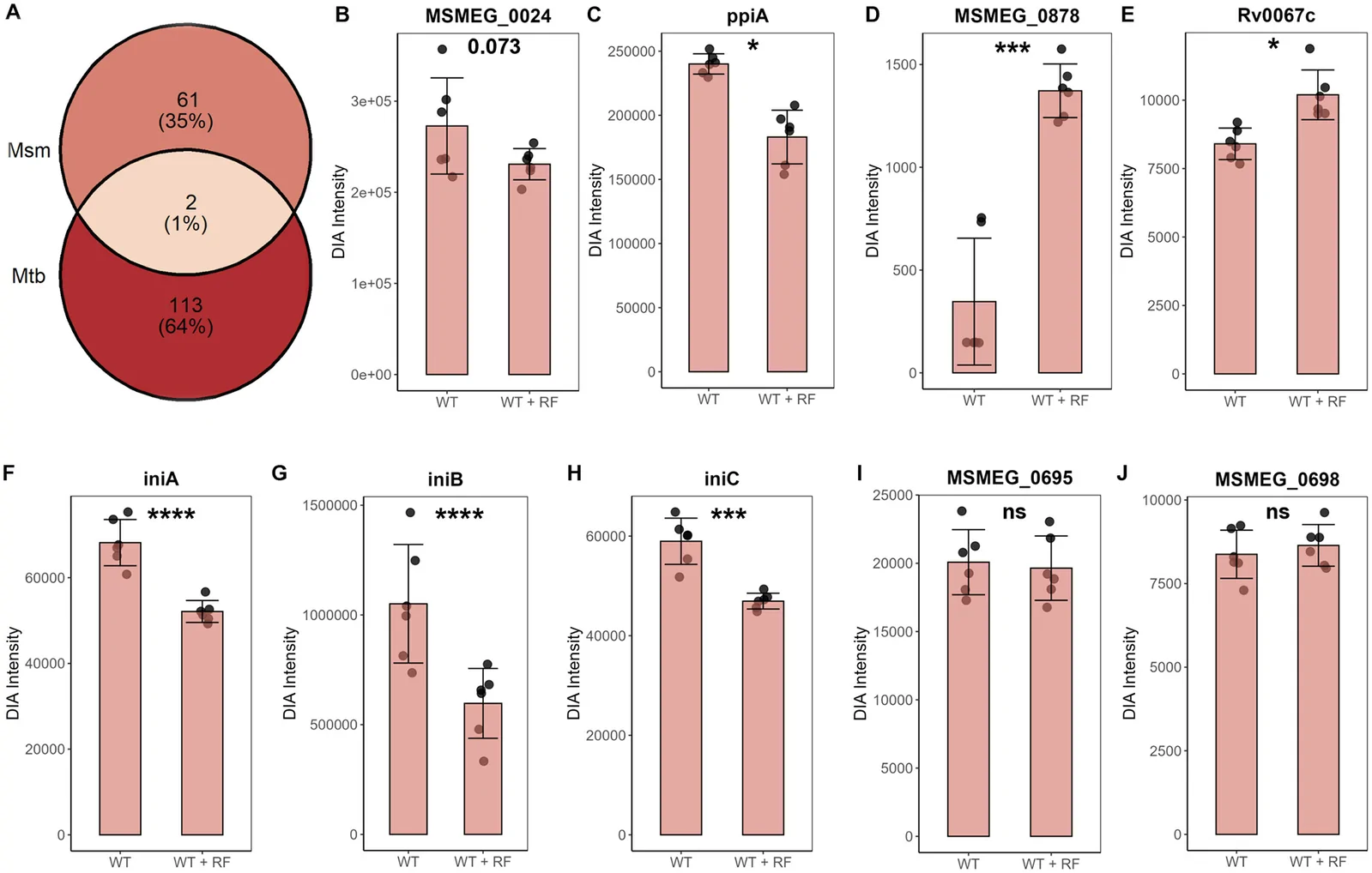
Elevated intracellular riboflavin differentially impacts isoniazid (INH) resistance factors in Msm and Mtb. **(A)** Overlap between features from Mtb and Msm selected using univariate analysis cutoff (*p*-value <0.05 and log2 Fold change >1) and first principal component of sPLS-DA. **(B, C)** Elevated intracellular riboflavin decreases abundance of PpiA in Mtb and Msm. Msm PpiA **(B)**, Mtb PpiA **(C)**. **(D, E)** Elevated intracellular riboflavin increases abundance of OxiR in Mtb and Msm. Msm OxiR **(D)**, Mtb OxiR **(E)**. **(F–J)** Elevated intracellular riboflavin decreases abundance of INH inducible factors in only Mtb. Mtb IniA **(F)**, Mtb IniB **(G)**, Mtb IniC **(H)**. Msm IniA **(I)**, Msm IniC **(J)**. Data are shown as mean quantification ± SD. Statistical comparison was performed using log transformed values followed by unpaired *t*-test and multiple test correction using Benjamini–Hochberg adjusted *p*-value. Statistical significance is represented by *p* < 0.05, *p* < 0.0005, *p* < 0.0001, shown by *, ***, ****, respectively. WT, Wild type; RF, riboflavin; ns/Not Sig., not significant.

To determine if other aspects of the proteomic response to elevated intracellular riboflavin are shared between Mtb and Msm, we examined homology among proteins prioritized by differential abundance analysis (*limma*) and multivariate feature selection (sPLS-DA). Of the 170 Msm proteins prioritized, 63 had homologs in Mtb. Intersecting these 63 homologs with the Mtb proteins selected by limma and sPLS-DA identified 2 shared candidates implicated in the riboflavin response (Figure 5A). The two proteins that overlapped were PpiA (MSMEG_0024, Rv0009) and OxiR (MSMEG_0878, Rv0067c). PpiA, peptidyl propyl isomerase, is a secreted protein which serves as a chaperone involved in stress adaptation while OxiR is a transcription factor whose overexpression induces resistance to isoniazid (INH) (31–33). PpiA had a reduced abundance (Figures 5B, C) while OxiR had an increased abundance (Figures 5D, E) in response to riboflavin in both organisms. Given the connection between OxiR and INH resistance, we checked if riboflavin treatment impacted any of the INH inducible genes. We observed a decrease in IniA (Figure 5F), IniB (Figure 5G) and IniC (Figure 5H) in response to riboflavin in Mtb. This trend was not observed in Msm for the homologs IniA (Figure 5I) or IniC (Figure 5J).

## Discussion

The unwavering nature of Mtb to currently available treatment and prevention strategies has been attributed to its ability to modulate its physiology in response to potentially toxic exposures. Although, the study of Mtb is more than a century old, there are still aspects of its physiology that remain enigmatic. Uncovering and understanding these aspects of its physiology is pertinent to Mtb eradication. Riboflavin, although an essential metabolite, has been shown to induce toxic effects at high concentrations in some microorganisms including bacteria and fungi that are capable of endogenous production (12–14). In a previous study, we observed that the growth rate of both Msm and Mtb were unaffected by increased intracellular concentrations of riboflavin (4, 5). This resistance indicated that mycobacteria may encode a mechanism to withstand the toxic effect of high riboflavin. However, none of the known mechanisms for flavin homeostasis has been reported in mycobacteria (5, 15). In our previous report (5), we showed the absence of transcriptional suppression of the RBP genes following treatment of Mtb and Msm with exogenous riboflavin. This likely indicates the absence of riboswitch-based regulation, a common mechanism for flavin homeostasis in bacteria, and instead suggests the presence of alternative regulatory mechanisms. To uncover the potential mechanism for flavin homeostasis used by mycobacteria, we use a bioinformatic approach to understand the changes in the proteome of Mtb and Msm in response to exogenous riboflavin based on data obtained through a DIA-PASEF based proteomics approach.

We previously showed that treatment with exogenous riboflavin led to a high intracellular level in both Msm and Mtb which we recapitulated using HRMS in this study (5).One of the reported mechanisms for flavin homeostasis is through the allosteric inhibition of RibF, the flavin kinase required to synthesize FMN and FAD (8). To determine if this was a possible mechanism in Mtb, we measured the intracellular level of FMN and FAD with or without riboflavin treatment. We observed that the levels of FMN and FAD did not reflect what was observed for riboflavin. Instead in Mtb, we observed a lower level of both coenzymes while Msm had similar levels regardless of the amount of riboflavin available. This result indicates the likelihood of a mechanism controlling the flux from riboflavin to downstream products. Since we observed an increase in the level of RibF when there was high levels of riboflavin, we hypothesize that this mechanism works post-translationally most likely through allosteric inhibition. The precise mechanism underlying the control of the flavin kinase activity of RibF will require further investigation.

Although FMN and FAD were not elevated, both Mtb and Msm presumably maintain systems to buffer the excess flavins through other flavin homeostatic mechanisms. Another mechanism of flavin homeostasis in mycobacteria is through the use of non-enzymatic carriers of flavin termed flavin sequesters. Flavin sequestration was first observed in archaea, a process involving dodecin, a small protein of 68 amino acids in length (34). It was observed that sequestration of flavin by dodecin both quenched their reactivity and ensured the availability of flavins during nutrient deficient situations (20, 35). These findings suggest two primary functions for flavin sequestering: maintaining flavin homeostasis and serving as a storage mechanism. The broad distribution and conserved nature of dodecin across archaea and eubacteria underscore its significance in these processes. In mycobacteria, the discovery of additional flavin sequesters, Fsq and Acg, further highlights its functional importance. From our proteomics data, we observed an increase in the abundance of Acg, dodecin and Fsq in response to elevated riboflavin concentrations in both Mtb and Msm, suggesting an adaptive response to sequester the excess flavin present. Fsq was first characterized in Msm, where its deficiency resulted in an inability to withstand oxidative stress during hypoxic growth (21). The role of Fsq in controlling oxidative stress during hypoxic growth may stem from the combined burden imposed by free flavins and reactive radicals on the stress response. This effect is likely amplified under hypoxia, as it is absent during normoxic growth. In the same study, an additional flavin sequestering protein (MSMEG_6368, Rv3129) was proposed based on sequence similarity to Fsq. However, MSMEG_6368 showed no change in abundance, and Rv3129 was not detected in our proteomic dataset (data not shown). Acg was initially proposed to function as a nitroreductase; however, functional assays revealed no detectable enzymatic activity (29). Despite lacking enzymatic activity, Acg was found to bind flavin, leading to the hypothesis that it functions as a flavin sequester. RHOCS has also been shown to be involved in flavin homeostasis by breaking down FAD (18). However, in contrast to the response observed for flavin sequesters, elevated riboflavin did not trigger a RHOCS response, consistent with findings from a previous study (18).

Interestingly, two of these flavin sequesters, Acg and Fsq, are controlled by DosR, a stress response transcription factor known for its role during dormancy in mycobacteria (21, 36). Since DosR is known to influence the transcription of other genes in Mtb and Msm, we further analyzed the proteome for additional changes that may be involved in flavin homeostasis. Using both a univariate and multivariate approach, we uncovered additional responses to elevated riboflavin. Although most of the proteins we identified were yet to be functionally characterized, an overwhelming number of the proteins were putatively characterized to be involved processes ranging from fatty acid metabolism, efflux/influx transportation, and transcription regulation based on our string analysis. Our STRING analysis for Msm showed that the proteins selected by sPLS-DA were involved in the response to stress and hypoxia, a response known to be coordinated by DosR (23, 24, 37). Using the TFOE dataset from ERGIN, we were able to attribute the response to excess riboflavin observed in Mtb to 8 TFs with 3 of these TFs regulating proteins with higher abundance in Mtb treated with riboflavin. By combining the ChIP-seq data and TFOE data from ERGIN, we observed that the proteomic changes in response to riboflavin may be a concerted effort to ensure flavin homeostasis. For DosR, the fold change in response to riboflavin correlated positively with the TFOE response for its regulatory targets in both Msm and Mtb. Furthermore, we observed an increase in the abundance of DosR in Msm and to a lesser extent in Mtb in response to elevated riboflavin. This evidence strongly suggests that the DosR regulated stress response is required for flavin homeostasis. For SigC, the correlation was negative as expected as our initial TFOE analysis showed that it upregulated proteins with lower abundance in Mtb treated with riboflavin. Although the homolog for SigC is absent in Msm, a similar trend was observed for the predicted regulatory targets in Msm (Data not shown). SigC has been implicated in coordinating the expression of genes involved in copper acquisition during scarcity (38). The reason for the observed downregulation of its regulatory targets in this context remains unclear. For OxyS, we observed opposing trends in Mtb and Msm, although none of its regulatory targets had a significant change in abundance. The role of OxyS in oxidative stress in mycobacteria appears to be less straightforward than that of DosR. In both Mtb and Msm, OxyS overexpression has been shown to increase susceptibility to oxidative stress (39–41). Although mutations and duplication of its ORF have been associated with INH resistance, experimental knockout or overexpression of OxyS did not alter susceptibility to INH (40). In other bacteria, OxyS has been shown to induce resistance to aminoglycosides during stress (42). The role of OxyS in this scenario may be beyond what is currently understood, and further investigation is needed to clarify its role.

Beyond the similarities and differences in the regulatory landscape, additional parallels were observed in the response to elevated riboflavin in Mtb and Msm. Overlapping the features from our univariate and multivariate analyses revealed that both PpiA and OxiR exhibited similar trends in response to elevated riboflavin, with PpiA showing reduced abundance and OxiR showing increased abundance. PpiA is a secreted protein that has been shown to chaperone proteins during cellular stress. Its role as a secreted protein has primarily been described in the context of pathogen–host interactions, where it binds to host integrin receptors, exacerbating disease and subverting host-induced stress (43, 44). Given its low cellular abundance in riboflavin treated samples, its primary function as part of the secretome may be involved in redox homeostasis. However, its secretion could simply be an automatic stress response, with no direct role in flavin homeostasis. OxiR, on the other hand, is a highly conserved TF in mycobacteria that belongs to the TetR/AcrR family, which is involved in INH resistance (31). Interestingly, we also observed a reduced abundance in IniA, IniB and IniC upon exposure to riboflavin in only Mtb. This dynamism in INH susceptibility factors and its impact on the susceptibility to INH in Mtb has yet to be clearly understood and will need further studies. The reason for the difference in the impact of elevated riboflavin on the INH inducible factors between Mtb and Msm additionally requires further clarification especially since Msm is naturally insensitive to INH (40, 45).

In conclusion, we showed that elevated riboflavin did not raise FMN or FAD levels in Msm or Mtb. This pattern is consistent with the allosteric inhibition of RibF, although sequestration of excess FMN and FAD by flavin-sequestering proteins could similarly reduce their measurable pools. Definitive studies are needed to determine if RibF can be allosterically inhibited or if sequestration is the primary mechanism to control the availability of FMN and FAD. Our analyses also showed that both Mtb and Msm mounted a coordinated change in their proteome in response to high riboflavin. This response seems to be coordinated by DosR, a well-studied stress response TF in mycobacteria. We also observed a marked change in the proteome of Msm in comparison to Mtb. However, this difference in response can be attributed to the lower concentration of riboflavin used to treat Mtb. It is also likely that Mtb responds differently to elevated riboflavin with other DosR independent mechanisms such as dodecin playing a significant role in flavin homeostasis. Interestingly, our data is also in congruence with the previous report of reduced FMN and FAD being strong activators of DosS, one of the histidine kinases that activates DosR (46). We therefore propose a model for flavin homeostasis in mycobacteria where excess reduced FMN and FAD are available to activate DosS and subsequently DosR. The corollary upregulation of the DosR regulon which includes the described flavin sequesters then leads to reduced levels of FMN and FAD. The achievement of flavin homeostasis then prevents the continued activation of DosS. Based on this model, the observation of no increase in the abundance of DosR in response to elevated riboflavin in Mtb reflects the achievement of flavin homeostasis by the timepoint selected for our analyses as the levels of FMN and FAD we observed were below those produced endogenously. It is also possible that the presence of DosR independent flavin sequesters such as dodecin in Mtb allows a more rapid achievement of flavin homeostasis. A further understanding of this model and other factors involved in flavin homeostasis, including studies to evaluate the kinetics of flavin homeostasis will be the subject of future studies. We also observed that elevated riboflavin influenced INH resistance factors, with both shared and distinct effects between in Msm and Mtb. For example, the induction of OxiR in both Mtb and Msm converse to the downregulation of INH inducible factors in only Mtb. We plan to investigate how these changes affect INH sensitivity in future studies. This work also opens several avenues for further exploration, including the link between riboflavin homeostasis and metal homeostasis. Notably, we observed potential connections with copper and iron regulation through MymT in Mtb and EccA3 in Msm, respectively. Studies in other organisms have shown regulatory interdependence between iron and riboflavin (47, 48).

Recently, vitamin C, was shown to increase the activity of other antimicrobials through the Fenton reaction in both *in vitro* and *in vivo* models (49, 50). Vitamin C on its own did not impact the viability of Mtb. These findings have made the supplementation of vitamin C intake a potential treatment strategy for Mtb (51). Vitamin C has also been shown to induce a dormancy response leading to growth stasis and reduced susceptibility to some antitubercular agents (52). However, vitamin C works synergistically with pyrazinamide, an antitubercular agent known to target dormant Mtb cells (52), this possibly negating antitubercular concerns. For riboflavin, aka vitamin B2, supplementation has also been considered as a therapeutic approach for other pathogens with some studies showing success (12–14). Unlike the response to vitamin C where Mtb goes into dormancy, the response to riboflavin seems to overcome the growth stasis as we have previously observed (4, 5). Given this, it will be necessary to test the impact of riboflavin-induced stress on susceptibility of Mtb to antimycobacterials. A clear understanding of this impact will be important for the consideration of riboflavin supplementation during Mtb infection. Furthermore, given that riboflavin biosynthesis is a recognized drug target, this work may provide insights into how its pharmacological inhibition and the availability of flavins influence susceptibility to other antimycobacterial agents.

## Data Availability

The datasets presented in this study can be found in online repositories. The names of the repository/repositories and accession number(s) can be found in the article/Supplementary material.
