# Supplementary material for: Proteomic insights into mycobacterial responses to elevated riboflavin reveal a role for flavin-sequestering proteins in flavin homeostasis
Source: Front Tuberc. 2025 Dec 17;3:1719707. doi: 10.3389/ftubr.2025.1719707 (PMC13494985; doi:10.3389/ftubr.2025.1719707)
Supplement: Supplementary file 1 [file Data_Sheet_1.docx]

**Proteomic Insights into Mycobacterial Responses to Elevated Riboflavin Reveal a Role for Flavin-Sequestering Proteins in Flavin Homeostasis**

**Nurudeen Oketade^1^, Karen M. Dobos^1*^**

^1^Department of Microbiology, Immunology and Pathology, Colorado State University, Colorado, United States of America

***Correspondence:**

Corresponding Author

[Karen.Dobos@colostate.edu](mailto:Karen.Dobos@colostate.edu) (K.M.D)

**Keywords**

Mycobacteria, Riboflavin, Flavin Sequesters, Proteomics, Tuberculosis, Isoniazid, Homeostasis, Stress Response

Supplementary Material

**S1 Figure: Quality Control for Proteomic Analysis of Mtb Samples.** **(A-B)** Distribution of percentage missingness of proteins before imputation **(A)** and after imputation **(B)**. Overall abundance of detected proteins in Mtb samples treated with or without riboflavin. Boxplots represent median and interquartile range. Coefficient of variation (CVs) for detected proteins between technical replicates **(D)** and biological replicates **(E)**. Vertical line showing point corresponding to 30% CV.

**S2 Figure:** **Quality Control for Proteomic Analysis of Msm Samples. (A-B)** Distribution of percentage missingness of proteins before imputation **(A)** and after imputation **(B)**. Overall abundance of detected proteins in Msm samples treated with or without riboflavin. Boxplots represent median and interquartile range. Coefficient of variation (CVs) for detected proteins between technical replicates **(D)** and biological replicates **(E)**. Vertical line showing point corresponding to 30% CV.

**S3 Figure: Impact of elevated intracellular riboflavin on abundance of RibF. (A-B)** Protein abundance was measured using DIA-PASEF proteomics (n = 3 biological replicates, 2 technical replicates). Elevated intracellular riboflavin increases abundance of RibF in Mtb and Msm. Msm RibF **(A)**, Mtb RibF **(B)**. Data are shown as mean quantification ± SD. Statistical comparison was performed using log transformed values followed by unpaired t-test and multiple test correction using Benjamini-Hochberg adjusted p-value. Statistical significance is represented by p<0.05, p<0.001, p<0.0005, p<0.0001, shown by *, **, ***, **** respectively. WT, Wild type; RF, riboflavin.

**S4 Figure: Detection of riboflavin, FMN, and FAD in Msm. (A-B)** Representative chromatograms depicting the precursor ion (MS^1^) for riboflavin, FMN and FAD in Msm. Each metabolite was monitored using Parallel Reaction Monitoring (PRM). Peaks are labelled with total peak area. **(B)** Collision-induced dissociation fragment ion spectra of *m/z* 377.1456 (riboflavin), *m/z* 457.1119 (FMN) and *m/z* 786.1644 (FAD) in Msm. RF, Riboflavin.

**S5 Figure: Detection of riboflavin, FMN, and FAD in Mtb. (A-B)** Representative chromatograms depicting the precursor ion (MS^1^) for riboflavin, FMN and FAD in Mtb. Each metabolite was monitored using Parallel Reaction Monitoring (PRM). Peaks are labelled with total peak area. **(B)** Collision-induced dissociation fragment ion spectra of *m/z* 377.1456 (riboflavin), *m/z* 457.1119 (FMN) and *m/z* 786.1644 (FAD) in Mtb. RF, Riboflavin.

**S6 Figure:** **Standards for riboflavin, FMN and FAD (A)** Representative chromatogram and **(B)** collision-induced dissociation fragment ion spectra of standards for *m/z* 377.1456 (riboflavin), *m/z* 457.1119 (FMN) and *m/z* 786.1644 (FAD). RF, Riboflavin.

**S7 Figure: STRING protein-protein interaction network for features selected by univariate and multivariate analyses. (A-B)** Protein-Protein interaction network of features selected by univariate analysis in Mtb **(A)** and Msm **(B)**. **(C-E)** Protein-Protein interaction network of features selected by multivariate analysis (sPLS-DA) in Mtb **(C)** and bar chart showing enrichment for local network clusters **(D)** and cellular component **(E)**. **(F-H)** Protein-Protein interaction network of features selected by multivariate analysis (sPLS-DA) in Msm **(F)** and bar chart showing enrichment for molecular function **(G)** and biological process **(H)**.

**S8 Figure: Heatmap showing Sparse Partial Least Square Discrimination Analysis (sPLS-DA) selected features. (A-B, D-E)** Heatmap showing the log2 abundance of proteins selected by sPLS-DA in Mtb **(A-B)** and Msm **(D-E)**. The color scale represents the scaled abundance per protein. **(C, F)** Overlap between proteins selected by both univariate (*limma)* and multivariate (sPLS-DA) analysis and flavoproteins in Mtb (C) and Msm (F) according to UniprotKB. Samples are aggregated based on hierarchical clustering. **(G-H)** Functional categories of proteins selected by sPLS-DA for Mtb **(G)** and Msm **(H)**.

**S9 Figure: Transcription factor profiling of proteome-wide change in response to elevated intracellular riboflavin in Mtb and Msm.** Pearson correlation between log2 fold change and TFOE expression change for regulatory targets (direct and operonic) of transcription factors based on ERGIN ChIP-seq data for Mtb **(A-E)** and Msm **(F-I)**. Correlation coefficient R and corresponding p-value shown. A significant correlation is observed for SigC (negative) **(B)** and OxyS (positive) **(E)** in Mtb. A significant correlation is observed for and OxyS (positive) **(I)** in Msm. Legend showing statistical significance of fold change based on cut-off for univariate analysis (p-value<0.05 and log2 Fold change >1). Not Sig., not significant.

**S1 Table. Window scheme used for DIA-PASEF acquisition**

| #MS Type | Cycle Id | Start IM [1/K0] | End IM [1/K0] | Start Mass [m/z] | End Mass [m/z] | CE [eV] |
| --- | --- | --- | --- | --- | --- | --- |
| MS1 | 0 | - | - | - | - | - |
| PASEF | 1 | 1.3806 | 1.4433 | 1385.82 | 1435.82 | - |
| PASEF | 1 | 1.2112 | 1.38 | 1091.82 | 1141.82 | - |
| PASEF | 1 | 0.9869 | 1.1987 | 748.82 | 798.82 | - |
| PASEF | 1 | 0.795 | 0.9617 | 503.82 | 553.82 | - |
| PASEF | 1 | 0.7101 | 0.7657 | 307.82 | 357.82 | - |
| PASEF | 2 | 1.4088 | 1.4433 | 1434.82 | 1484.82 | - |
| PASEF | 2 | 1.2394 | 1.4039 | 1140.82 | 1190.82 | - |
| PASEF | 2 | 1.0234 | 1.2305 | 797.82 | 847.82 | - |
| PASEF | 2 | 0.8334 | 1.0106 | 552.82 | 602.82 | - |
| PASEF | 2 | 0.7063 | 0.8147 | 356.82 | 406.82 | - |
| PASEF | 3 | 1.2959 | 1.4433 | 1238.82 | 1288.82 | - |
| PASEF | 3 | 1.0949 | 1.2844 | 895.82 | 945.82 | - |
| PASEF | 3 | 0.8718 | 1.0596 | 601.82 | 651.82 | - |
| PASEF | 3 | 0.7183 | 0.8637 | 405.82 | 455.82 | - |
| PASEF | 4 | 1.183 | 1.3561 | 1042.82 | 1092.82 | - |
| PASEF | 4 | 0.9485 | 1.1576 | 699.82 | 749.82 | - |
| PASEF | 4 | 0.7567 | 0.9127 | 454.82 | 504.82 | - |
| PASEF | 5 | 1.3241 | 1.4433 | 1287.82 | 1337.82 | - |
| PASEF | 5 | 1.1266 | 1.3083 | 944.82 | 994.82 | - |
| PASEF | 5 | 0.9101 | 1.1086 | 650.82 | 700.82 | - |
| PASEF | 6 | 1.2677 | 1.4278 | 1189.82 | 1239.82 | - |
| PASEF | 6 | 1.0591 | 1.2604 | 846.82 | 896.82 | - |
| PASEF | 7 | 1.3523 | 1.4433 | 1336.82 | 1386.82 | - |
| PASEF | 7 | 1.1548 | 1.3322 | 993.82 | 1043.82 | - |

**S2 Table. Precursors and transition ions used for targeted metabolomics by MRM-MS**

| **Compound** | **Abbreviation** | **Precursor m/z** | **Transitions m/z** | **Retention Time (Min)** |
| --- | --- | --- | --- | --- |
| Riboflavin | RF | 377.1456 | 172.08 | 7.1 |
|  |  |  | 198.06 |  |
|  |  |  | 243.08 |  |
|  |  |  | 359.13 |  |
| Flavin mononucleotide | FMN | 457.1119 | 243.08 | 6.5 |
|  |  |  | 359.13 |  |
|  |  |  | 396.09 |  |
|  |  |  | 439.09 |  |
| Flavin adenine dinucleotide | FAD | 786.1644 | 136.06 | 6.1 |
|  |  |  | 250.09 |  |
|  |  |  | 307.37 |  |
|  |  |  | 348.06 |  |
